# Neuropsychiatric symptoms as risk factors of dementia in a Mexican population: A 10/66 Dementia Research Group study

**DOI:** 10.1016/j.jalz.2017.08.015

**Published:** 2018-03

**Authors:** Isaac Acosta, Guilherme Borges, Rebeca Aguirre-Hernandez, Ana Luisa Sosa, Martin Prince

**Affiliations:** a Laboratory of Dementias, National Institute of Neurology and Neurosurgery, Mexico City, Mexico; b Departament of Epidemiologic and Psychosocial Research, National Institute of Psychiatry Ramon de la Fuente Muniz, Mexico City, Mexico; c Department of Pharmacology, School of Medicine, National Autonomous University of Mexico, Mexico City, Mexico; d Department of Health Service and Population Research, King's College London (Institute of Psychiatry), London, UK

**Keywords:** Neuropsychiatric symptoms, Risk factors, Dementia, Aged, Population-based study

## Abstract

**Introduction:**

Cognitive and/or memory impairment are the main clinical markers currently used to identify subjects at risk of developing dementia. This study aimed to explore the relationship between the presence of neuropsychiatric symptoms and dementia incidence.

**Methods:**

We analyzed the association between neuropsychiatric symptoms and incident dementia in a cohort of 1355 Mexican older adults from the general population over 3 years of follow-up, modeling cumulative incidence ratios using Poisson models.

**Results:**

Five neuropsychiatric symptoms were associated with incident dementia: delusions, hallucinations, anxiety, aberrant motor behavior, and depression. The simultaneous presence of two symptoms had a relative risk, adjusted for mild cognitive impairment, diabetes, indicators of cognitive function, and sociodemographic factors, of 1.9 (95% confidence interval, 1.2–2.9), whereas the presence of three to five, similarly adjusted, had a relative risk of 3.0 (95% confidence interval, 1.9–4.8).

**Discussion:**

Neuropsychiatric symptoms are common in predementia states and may independently contribute as risk factors for developing dementia.

## Highlights

• Not only cognitive impairment and cognitive features are useful in identifying older adults at risk for developing dementia.
• Of the 12 neuropsychiatric symptoms evaluated, 5 were associated with incident dementia (delusions, hallucinations, depression, anxiety, and aberrant motor behavior) in a 3-year follow-up.
• The evaluation of neuropsychiatric symptoms, along with other simple, practical, and inexpensive features, may be useful in identifying subjects at risk of dementia in community health settings and by general practitioners.

## 1 Introduction

In 2015, it was estimated that there were 46.8 million people with dementia worldwide, of whom 58.0% were living in low- and middle-income countries [1], and that there were annually 9 million new global cases (2014–2015) of dementia. In response to the high prevalence of dementia, the aging global population (8%–9% of the population aged 60 years or older), and its important socioeconomic impact, the World Health Organization made dementia a “public health priority” that requires immediate action [2]. While it has been estimated that if dementia care were a country, it would be the world's 18th largest economy [1], it has become clear that there is a significant imbalance in the global distribution of the quantity and quality of the resources available to treat this illness.

Because a curative treatment is not available so far, several recent lines of research have focused on looking for markers (clinical or biological) that may allow subjects at risk of developing dementia to be identified [3], [4], [5], [6] to undertake timely interventions to modify the course of the disease or delay its progress [7]. Neuropsychiatric symptoms (NPSs) have been proposed as potential clinical markers [8], [9], [10] for dementia, and it has been considered that some of them, such as depressive and anxiety symptoms, may increase the risk of developing dementia [11], [12], [13], [14], [15]. This has been documented even after controlling for known sociodemographic, genetic, cognitive, and metabolic (diabetes) risk factors [16], [17]. However, evidence for the capacity of NPSs to predict the onset of dementia is still inconclusive [18]. The diversity in the results in this field can be partly explained by differences among the studies in terms of the populations, follow-up time, control of risk factors, assessment and diversity of the NPSs, and a range of other factors. In addition, this evidence has been generated in high-income countries, and their study in low- and middle-income countries is limited.

Given the speed of population aging in our country, and the urgent need of markers for preclinical identification of dementia, this report aimed to analyze the relationship between the presence of NPSs and dementia incidence, testing their capability to identify subjects at risk of dementia, through a 3-year follow-up study of Mexican older adults from the general population.

## 2 Methods

### 2.1 Sample and procedure

Our report comprised information about 1823 adults aged 65 years and older, living in urban and rural zones, without dementia diagnosis at baseline evaluation (2003–2006), followed in accordance with the 10/66 Dementia Research Group (DRG 10/66) protocols. All the participants were contacted for a 3-year follow-up interview and evaluation (2007–2010). The details of the selection, recruitment, and follow-up method of the cohort have been described in detail elsewhere [19], [20], [21].

The urban zones selected for the recruitment were located in the south of Mexico City, and rural recruitment was done in the municipalities of Huitzilac and Tepoztlan, in the state of Morelos. Participants were identified through a door-to-door census, with a response rate at baseline of 85.1%. In both phases (baseline and follow-up), the following assessments were applied: (1) household questionnaire, (2) cognitive evaluation, (3) semistructured geriatric mental state interview, (4) sociodemographic and risk factors questionnaire, (5) an informant or principal caregiver interview (who was close to the older adult), and (6) general physical assessment and blood extraction for clinical analysis (mainly blood cell count and chemistry). Evaluations were performed by Psychology and Social Work undergraduates, and physicians trained at the National Institute of Neurology and Neurosurgery. All the evaluations were based on the DRG 10/66 manuals and training sessions. Participants signed an informed consent; illiterate participants provided verbal consent in the presence of a witness. The study was approved by the scientific and ethical committees of the National Institute of Neurology, Mexico and for the King's College London, United Kingdom [21].

### 2.2 Measurements

#### 2.2.1 Dementia

We established a dementia diagnosis according to 10/66 and the Diagnostic and Statistical Manual of Mental Disorders IV criteria. The algorithms to operationalize these criteria were developed by the 10/66 DRG and have been reported and described elsewhere [22], [23]. Briefly, 10/66 dementia cases score above a cutoff point of predicted probability for dementia based on cognitive test, informant report scores, and diagnostic output from clinical interviews [22]. Diagnostic and Statistical Manual of Mental Disorders IV dementia cases must meet all four qualifying criteria: (1) characteristic cognitive impairment, (2) decline in social or occupational functioning, (3) not accounted for by another mental disorder, and (4) not occurring only during delirium [23]. These algorithms were validated in population samples, having as gold standard the diagnosis made by specialist doctors [23].

#### 2.2.2 Neuropsychiatric symptoms

The questionnaire version of the Neuropsychiatric Inventory (NPI-Q) [24] was used to assess NPSs. The NPI-Q is a structured interview that is applied to the caregiver or an informant close to the older adult and collects information on the presence of the 12 most common symptoms in patients with dementia, during the previous month: delusions, hallucinations, agitation/aggression, depression, anxiety, euphoria, apathy, disinhibition, irritability, aberrant motor behavior, and eating and sleep disorders. In this study, we considered the presence/absence of each one of the 12 NPSs evaluated, analyzing their prevalence and their association with incident dementia.

#### 2.2.3 Other variables

##### 2.2.3.1 Mild cognitive impairment

This was diagnosed in compliance with the Mayo Clinic criteria, again using an algorithm developed by DRG 10/66, which considers (1) subjective complaints regarding memory, (2) slight impairment in cognitive tasks, (3) preservation of functionality for daily activities, and (4) absence of dementia; those who met the criteria were classified as mild cognitive impairment (MCI) cases [25].

##### 2.2.3.2 Diabetes mellitus type 2

Diabetes mellitus type 2 was diagnosed if (1) fasting glycemia at baseline was ≥126 mg/dL or (2) the older adult reported having been diagnosed as diabetic by a health professional [26].

##### 2.2.3.3 Disability

We classified participants as disabled based on a score equal to or above the 90th percentile of the World Health Organization disability scale whose psychometric properties have been described elsewhere [27].

##### 2.2.3.4 Cognitive function

We selected three indicators of cognitive function. The first two refer to executive functioning and the latter to cognitive reserve: (1) verbal fluency—assessed using the 60-second semantic verbal fluency test (“animals” category) considering impairment when the score was ≤1.5 standard deviation adjusted for age group and level of educational attainment; (2) simple motor tasks—assessed using the Luria three-motor sequencing test (fist-edge-palm), failure in this test was indicated when the participant was unable to carry out five continuous correct sequences; and (3) illiteracy—evaluated by self-reporting the inability to read the newspaper [20].

##### 2.2.3.5 Sociodemographic variables

The following variables were collected: (1) age (years); (2) gender (male or female); (3) level of educational attainment (none, basic education complete or incomplete, and secondary or tertiary schooling); (4) catchment area (urban or rural); (5) assets/services in the home (car, television, refrigerator, telephone, drinking water, mains drainage and electricity) and food insecurity, indicated by self-reporting of being hungry in the past month due to lack of food. This information was taken from the household and sociodemographic questionnaires [21].

### 2.3 Statistical analysis

To assess the differences between the participants with and without longitudinal follow-up because of attrition (refusal, deaths, and nonlocatable participants), summary statistics, chi-square tests, and *t* tests (for continuous age) were calculated for sociodemographic variables (age, gender, level of educational attainment), area of study (urban/rural), and MCI diagnoses.

The distribution of sociodemographic, cognitive function measures, and clinical variables (including NPSs) among incident dementia cases and noncases was computed after adjusting for household clustering [20]. The incidence rate of dementia (per 1000 person-years) among these variables was estimated by dividing the number of cases by the person-years in each group. Person-years at risk for the onset of dementia was calculated as the period between baseline and follow-up assessment, using the midpoint of this interval for those who developed dementia [20], given the difficulty of establishing a more precise onset time of dementia.

For evaluating the association between incident dementia and baseline NPSs, cumulative incidence ratios were estimated using Poisson regression models with robust confidence intervals at 95% level [28], [29]. This procedure is used for estimating the relative risk (RR) when the outcome is a dichotomous variable [30] and provides estimates similar to hazard ratios using the Cox proportional hazards model when the follow-up time has been censored [31]. Given the correlation between some NPSs, we analyzed the independent effect of each NPS and their combined impact to select the symptoms with the best potential as predictors of dementia. To select the best approach, we carried out our analyses and selection models in five steps: (1) we tested the association of each NPS with incident dementia individually in adjusted models (that included age, gender, level of educational attainment, and MCI); (2) we created one model with the 12 symptoms mutually adjusted; (3) we developed one model with a count variable (from 0 to 12) with each NPS, and it was classified in three levels: (i) absence of symptoms or up to one NPS, (ii) two NPSs, and (iii) three or more NPSs; (4) we created another model including only those symptoms that had a *P* value of ≤0.15 and which were statistically significant in NPS independent effects models; (5) after selecting significant NPSs (*P* value ≤ .15 in models of independent effects), we created another categorical count variable with the same three levels described previously: (i) absence or up to one NPS, (ii) two NPSs, and (iii) three or more. As a last approach, categorical count variable with significant NPSs in models of independent effects was adjusted by the presence of known risk factors, to assess the impact of sociodemographic, clinical, and cognitive function variables on the categorical count variable estimations. For selecting models with the best fit, we considered the values of Bayesian and Akaike information criteria (the latter as a predominant criteria) [31], [32] in the inclusion of NPSs (categorical count variable with significant NPSs) and to adjust this model with sociodemographic, clinical, and cognitive function variables.

Finally, we developed a composite index by the number of NPSs and other risk factors, as an initial proposal to identify subjects at risk of developing incident dementia. We tested this index and its ability to correctly classify participants who did and did not develop dementia, using various cutoff points. For each cutoff point, we compared the predictive ability with dementia diagnoses, using sensitivity, specificity, positive and negative predictive values, and the total area under the ROC curve [23]. Similar analyses were performed in reduced indexes, removing MCI and NPSs one at a time, to assess how much the index's performance varied without each of these clinical indicators. All the analyses were performed using Stata 13.1 [33].

## 3 Results

### 3.1 Follow-up and losses

From the 1823 older adults without dementia evaluated at baseline, 1355 (74.3%) were re-evaluated at follow-up; 166 (9.1%) had died and 302 (16.6%) were lost (by refusal to take part, changing residence or being impossible to contact). The follow-up of 1355 participants resulted in a total of 3966.1 person-years. The average follow-up period was 2.9 years and the median was 3.0; the lower and higher quartile was 3.0 and 3.2 years, respectively.

Table 1 shows the sociodemographic characteristics of those who were re-evaluated and those who were lost in the follow-up. Those lost during follow-up were on average 1.6 years older (*P* < .001) than those re-evaluated; the percentage of men (*P* = .015) and the percentage of participants living in rural areas (*P* = .019) were also greater among those lost. Both groups had a similar distribution by educational attainment and MCI diagnoses (Table 1). We also inspected the distribution of NPSs; the distribution was similar in 11 of the NPSs (*P* > .05), with the exception of anxiety, with 17.5% in followed-up individuals and 13.5% in those lost (*P* = .044) (data not shown).

**Table 1:** Sociodemographic and mild cognitive impairment conditions, by follow-up status

Variable	Re-interviewed	Losses	*P* value
	*n* = 1355	*n* = 468	
Age years, mean (standard error)	73.2 (0.17)	74.8 (0.32)	<.001
Age group %			
68–72	30.6	26.3	<.001
73–77	31.9	25.6	
78–82	20.7	22.4	
83+	16.8	25.6	
Female gender %	64.3	58.3	.015
Level of educational attainment %			
None	22.8	27.3	.157
Basic education (complete or incomplete)	67.0	62.4	
Secondary or tertiary	10.2	10.3	
Rural catchment area %	48.3	55.1	.019
Mild cognitive impairment %	3.1	3.6	.572

### 3.2 Incidence rate

The dementia incidence was 32.5 cases per 1000 person-years of follow-up. Higher rates of incidence were observed among older participants; women; low level of educational attainment (none or basic); those living in rural areas (with less assets and suffering from food insecurity); and those with MCI diagnoses, disability, diabetes, illiteracy, impaired semantic verbal fluency, or who were unable to complete the Luria motor sequence. For the NPSs, the highest incidence rates of dementia were among participants with hallucinations (93.0), delusions (75.8), aberrant motor behavior (71.0), anxiety (63.2), disinhibition (47.0), apathy (46.4), and depression (44.9) (Table 2).

**Table 2:** Sociodemographic variables, cognitive reserve measures, clinical status, and neuropsychiatric symptoms, distribution by incident versus nonincident dementia cases

Variable	Nonincident dementia cases, % (*n*)	Incident dementia cases, % (*n*)	Total, % (*n*)	Incidence rate (per 1000 person-years)
Age groups				
68–72	32.9 (404)	7.8 (10)	30.6 (414)	7.9
73–77	32.3 (396)	27.9 (36)	31.9 (432)	28.5
78–82	19.7 (241)	31.0 (40)	20.7 (281)	50.0
83+	15.1 (185)	33.3 (43)	16.8 (228)	68.0
Gender				
Male	36.0 (441)	33.3 (43)	35.7 (484)	30.0
Female	64.0 (785)	66.7 (86)	64.3 (871)	33.7
Level of educational attainment				
None	20.8 (254)	42.2 (54)	22.8 (308)	62.4
Basic (complete or incomplete)	68.5 (838)	52.3 (67)	67.0 (905)	25.0
Secondary or tertiary	10.7 (131)	5.5 (7)	10.2 (138)	16.9
Catchment area				
Urban	53.4 (655)	34.9 (45)	51.7 (700)	21.6
Rural	46.6 (571)	65.1 (84)	48.3 (655)	44.7
Assets and food insecurity				
>3 assets, no food insecurity	79.2 (967)	60.9 (78)	77.4 (1045)	25.2
>3 assets, food insecurity	3.7 (45)	4.7 (6)	3.8 (51)	41.2
≤3 assets, no food insecurity	14.9 (182)	28.1 (36)	16.2 (218)	58.4
≤3 assets, food insecurity	2.2 (27)	6.3 (8)	2.6 (35)	82.5
Mild cognitive impairment (MCI)	2.7 (33)	7.0 (9)	3.1 (42)	77.5
Disability (≥90 percentile in World Health Organization disability scale II)	5.1 (62)	10.1 (13)	5.5 (75)	62.8
Diabetes	24.1 (267)	35.3 (41)	25.2 (308)	46.6
Illiteracy	19.4 (236)	46.1 (59)	21.9 (295)	72.2
Semantic verbal fluency impaired	2.8 (34)	7.0 (9)	3.2 (43)	76.9
Less than 5 sequences in Luria's sequence (fist-edge-palm)	84.7 (1029)	93.7 (120)	85.5 (1149)	35.8
Neuropsychiatric symptoms				
Delusions	6.9 (84)	17.1 (22)	7.8 (106)	75.8
Hallucinations	3.2 (39)	10.1 (13)	3.8 (52)	93.0
Agitation/aggression	14.8 (181)	18.6 (24)	15.1 (205)	40.9
Depression	31.6 (387)	44.2 (57)	32.8 (444)	44.9
Anxiety	15.9 (195)	32.6 (42)	17.5 (237)	63.2
Euphoria	2.1 (26)	2.3 (3)	2.1 (29)	35.5
Apathy	8.6 (105)	12.4 (16)	8.9 (121)	46.4
Disinhibition	5.8 (71)	8.5 (11)	6.1 (82)	47.0
Irritability	23.8 (292)	30.2 (39)	24.5 (331)	40.9
Aberrant motor behavior	3.0 (37)	7.0 (9)	3.4 (46)	71.0
Sleep disorders	25.1 (308)	29.5 (38)	25.5 (346)	38.0
Eating disorders	14.9 (182)	17.1 (22)	15.1 (204)	37.3
Total	100.0 (1226)	100.0 (129)	100.0 (1355)	32.5

### 3.3 Association between neuropsychiatric symptoms and incident dementia

Poisson models showed statistically significant individual associations for five NPSs with the incidence of dementia, which were as follows: hallucinations RR = 2.8 (95% CI 1.7–4.6), delusions RR = 2.4 (95% CI 1.6–3.7), anxiety RR = 2.3 (95% CI 1.6–3.2), aberrant motor behavior RR = 2.1 (95% CI 1.2–3.9), and depression RR = 1.6 (95% CI 1.2–2.3) (Table 3). After selecting the best adjust and independent effects, these five NPSs still remained associated, even when they were adjusted, but the RR was reduced in the multiple adjusted models (mutually adjusted effect column, Table 3). These adjusted RR ranged from 1.7 (anxiety and aberrant motor behavior) to 1.3 (hallucinations). A count variable was developed from these five identified NPSs at baseline (range 0–5) which resulted in the following crude RR for incident dementia for three categories: (1) 0–1 (reference); (2) 2 NPS: RR = 2.5 (95% CI = 1.7–3.7); (3) ≥3 NPS: RR = 3.8 (2.4–5.9) (data not shown).

**Table 3:** Independent and mutually adjusted effect of NPS as risk factors of dementia

Neuropsychiatric symptoms	Independent effect	Mutually adjusted effect∗
Delusions	2.4 (1.6–3.7)	1.6 (1.0–2.4)
Hallucinations	2.8 (1.7–4.6)	1.3 (0.8–2.2)
Agitation/aggression	1.3 (0.8–1.9)	
Depression	1.6 (1.2–2.3)	1.4 (1.0–2.0)
Anxiety	2.3 (1.6–3.2)	1.7 (1.2–2.5)
Euphoria	1.1 (0.4–3.1)	
Apathy	1.4 (0.9–2.3)	
Disinhibition	1.5 (0.8–2.6)	
Irritability	1.4 (0.9–1.9)	
Aberrant motor behavior	2.1 (1.2–3.9)	1.7 (1.0–3.1)
Sleep disorders	1.2 (0.9–1.7)	
Eating disorders	1.2 (0.7–1.8)	

Abbreviations: NPS, neuropsychiatric symptom; MCI, mild cognitive impairment.

NOTE. All estimators were adjusted by age, gender, level of educational attainment, and MCI.

∗ Model mutually adjusted by delusions, hallucinations, depression, anxiety, and aberrant motor behavior (NPSs had a *P* value ≤ .15, once the 12 symptoms were mutually adjusted).

We explored the impact of this count variable in fully adjusted models to predict the incidence of dementia with other risk factors (see model descriptions in Table 4). The model with the best value, selected using the Bayesian and Akaike information criteria, was the one that simultaneously considered our proposed count variable plus age, gender, catchment area, assets and food insecurity, MCI, diabetes, illiteracy, and impairment of verbal fluency (model 5, Table 4). When we compared this model with individuals with zero or one NPS, the presence of two NPSs increased the incidence of dementia with an RR = 1.9 (95% CI 1.2–2.9), and in cases with three or more symptoms, it resulted in an RR = 3.0 (95% CI 1.9–4.8).

**Table 4:** Dementia risk by neuropsychiatric symptoms count (0–5) by different Poisson models (adjusted by sociodemographic, clinical, and cognitive reserve variables)

Number of neuropsychiatric symptoms¶	Model 1∗	Model 2 (model 1 + MCI)	Model 3 (model 1 + MCI, diabetes, and disability)	Model 4 (model 1 + MCI and cognitive reserve variables†)	Model 5‡	Model 6§
0–1	1.0	1.0	1.0	1.0	1.0	1.0
2	2.1 (1.4–3.1)	2.1 (1.4–3.1)	1.9 (1.2–3.0)	2.1 (1.4–3.1)	1.9 (1.2–2.9)	1.9 (1.2–2.8)
3–5	3.3 (2.2–4.9)	3.3 (2.2–5.0)	3.1 (2.0–4.8)	3.0 (1.9–4.7)	3.0 (1.9–4.8)	3.0 (1.8–4.7)
Akaike information criteria	769.702	766.773	687.620	747.864	676.336	678.242
Bayesian information criteria	837.356	839.631	769.207	836.080	752.799	729.267

Abbreviation: MCI, mild cognitive impairment.

∗ Model 1 was adjusted by gender, education, catchment area, assets, and food insecurity.

† Cognitive reserve variables: illiteracy, impairment in verbal fluency, and impairment in Luria's motor sequence.

‡ Model 5 was adjusted by gender, age, catchment area, assets and food insecurity, MCI, diabetes, illiteracy, and impairment in verbal fluency.

§ Model 6 was adjusted by age, catchment area, MCI, diabetes, and illiteracy.

¶ The neuropsychiatric symptoms included delusions, hallucinations, anxiety, aberrant motor behavior, and depression.

**Fig. 1:**
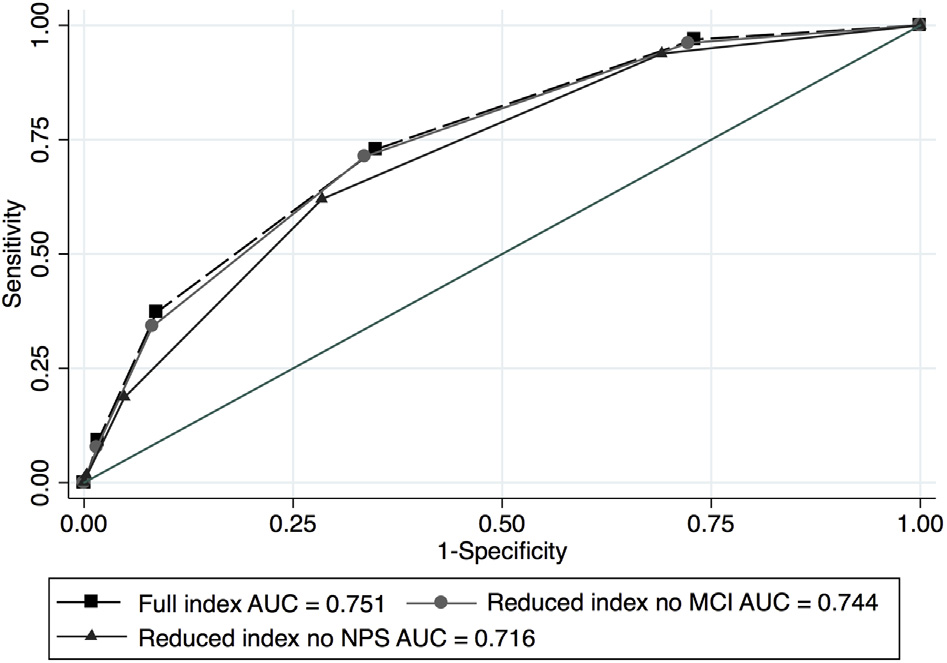
ROC curves for full index (range 0–6), reduced index without MCI (range 0–5), and reduced index without NPS (range 0–5). Abbreviations: AUC, total area under the receiver operating characteristic curve; MCI, mild cognitive impairment; NPS, neuropsychiatric symptom; PNV, predictive negative value; PPV, predictive positive value.

Finally, using only those statistically significant variables identified in model 5 (Table 4), we developed an index to identify the participants at risk of dementia that included the following: (1) age 80+, (2) living in a rural area, (3) MCI, (4) diabetes, (5) illiteracy, and (6) 2 or more than 2 NPSs, assigning a 0 for absence and a 1 for presence of each one of those six variables. The utility of this index (range 0–6) for predicting the incidence of dementia was estimated for various cutoff points (2, 3, and 4 risk factors present), among which, with a cutoff point of 2 (presenting two or more of the risk categories mentioned), the sensitivity value was 72.9%, the specificity 65.1%, with a positive predictive value of 18.0%, and a negative predictive value of 95.8% (Table 5). No differences were found when we compared the total area under the ROC curve for the full index versus the index without MCI (0.751 and 0.744, respectively; *P* = .209). When we compared the full index versus the index without NPSs, the area under the ROC curve decreased significantly from 0.751 to 0.716 (*P* = .001) (Fig. 1). A further comparison between the areas under the ROC curve for indexes without MCI versus without NPSs was also significant (*P* = .005).

**Table 5:** Accuracy of dementia classification, full and reduced indexes, 10/66 study Mexico follow-up

Index and AUC	Cutoff point	Sensitivity	Specificity	PPV	PNV
Full (count variable from 0 to 6) AUC = 0.751∗	2	72.9	65.1	18.0	95.8
		(64.3–80.3)	(62.3–67.8)	(14.8–21.6)	(94.2–97.1)
Reduced, without MCI (count variable from 0 to 5) AUC = 0.744†	2	71.3	66.4	18.3	95.7
		(62.7–78.9)	(63.7–69.0)	(15.0–21.9)	(94.1–96.9)
Reduced, without NPS (count variable from 0 to 5) AUC = 0.716‡	2	62.0	71.5	18.6	94.7
		(53.1–70.4)	(68.9–74.0)	(15.1–22.7)	(93.1–96.1)

Abbreviations: PPV, predictive positive value; PNV, predictive negative value; AUC, total area under the receiver operating characteristic curve; MCI, mild cognitive impairment; NPS, neuropsychiatric symptom.

NOTE. NPSs included delusions, hallucinations, anxiety, aberrant motor behavior, and depression.

∗ Count variable was performed with the sum of age 80+, live in rural area, MCI, diabetes, illiteracy, and 2 or more than 2 NPSs.

† Count variable was performed with the sum of age 80+, live in rural area, diabetes, illiteracy, and 2 or more than 2 NPSs.

‡ Count variable was performed with the sum of age 80+, live in rural area, diabetes, illiteracy, and MCI.

## 4 Discussion

This study concluded that 5 of the 12 NPSs evaluated with the NPI-Q (delusions, hallucinations, anxiety, aberrant motor behavior, and depression) were independent predictors of incident dementia in a 3-year follow-up of Mexican older adults from the general population. Our results agree with previous reports [18], [34], [35], [36], [37], which showed the usefulness of certain NPSs as risk factors in the preclinical stages of dementia [5], [38], [39], [40], [41], [42]. Of the five symptoms that proved to be linked to incident dementia, depression and anxiety are the ones that have been most consistently reported in the literature [18], [34], [37], [38], [40], [42], [43]. On the other hand, the association of incident dementia with aberrant motor behavior has only been reported in a few cross-sectional studies [44], [45] and in one follow-up study [34]. Regarding delusions and hallucinations, these are psychotic symptoms that are usually more prevalent in advanced stages of dementia [46] but they could be present in predementia stages, or be part of a depressive episode, psychotic syndrome or as isolated psychotic symptoms with late onset. Their presence in this population could also be explained by the “virgin state for treatments” of the participants, as both diagnosis and interventions (pharmacological and nonpharmacological) were almost nonexistent in our sample [7], [38].

Each of the five symptoms associated with the incident dementia had similar RRs and the increase in the number of NPSs elevated the probability of developing dementia. In the models used to estimate the strength of association of symptoms, we used as a reference category no or one symptom due to the high frequency of concurrent NPSs (e.g., depression and sleep disorders) as suggested elsewhere [18].

Our results in this sample of Mexican older adults are comparable to those in studies in developed countries. Edwards et al. [47] reported that the presence of more NPSs raised the risk of developing dementia among patients with MCI, and even more in those with amnestic MCI. Some reports support the possibility that NPSs could represent early manifestations in preclinical stages of dementias [38], [42]. If so, the presence of NPSs could help in the early identification of subjects at risk of developing dementia and the application of more timely interventions.

The increasing efforts to characterize predementia early stages involve complex elements including, on one hand, conceptual and ethical issues, competency questions, discrimination and stigma, and on the other hand, the possibility of offering therapies for specific symptoms such as anxiety, sleep problems, as well as the management of comorbidities. It is difficult to determine whether biological, cognitive, or behavioral markers must be studied per se, as they may represent prodromes or risk factors of dementia. In either case, the discussion is on the establishment of either timely diagnosis or early diagnosis, that is, between the onset of neuropathology, reliable predictive biomarkers, and the onset of cognitive decline symptoms [48]. At the moment, there is no clear consensus on whether these manifestations should be considered as risk factors, as prodromal or as early stages in the development of dementia.

The low prevalence and heterogeneity of MCI in the DRG 10/66 centers, including Mexico, has caught our attention. Our conclusion in this respect has been that this construct may be culturally affected [25]. The dementia incidence rate that we found is similar to that reported in other Mexican population studies [49], but it may appear high when it is compared with a recent report that includes a large Hispanic-Mexican sample [50]. In prior work by the 10/66 DRG group, a wide range in the incidence of dementia for the Latin American region was reported, with the Mexican incidence at a midpoint [20], [23], [51]. It is not clear at the moment the reasons that could be behind such variability in rates of dementia across populations.

The index developed in this work to identify people at risk of dementia seeks to provide a simple, practical, and inexpensive approach to be applied in the clinical field [52].

Before recommending this index for clinical purposes or as a diagnostic tool, its psychometric properties must be improved because of the low PPV of this index. One of the aims of this study is to emphasize the importance of general practitioners and geriatric health workers being more aware of the importance of identifying subjects at risk of developing dementia.

In relation to the properties of our index, its performance, although inferior to that accepted for diagnostic screening studies, is in line with the results of other studies that present predictive proposals for dementia [52], which report values of sensitivity and specificity less than 0.80. However, the stronghold of our index are the following: (1) it tested the simultaneous contribution of several NPSs as predictors of dementia in a wide range of ages (>65 years) and not only among the very old (>75 years), (2) the NPSs were tested directly in respect to other consistent predictive variables (i.e., cognitive performance and MCI), and (3) the variables included in this index can be measured easily (not requiring administration of specific neuropsychological tests) and inexpensively (not including expensive biomarkers). The study of the performance of this index should improve its properties and be expanded to other populations, in population-based studies and in general clinical practice [10].

One limitation is that our data are not nationally representative; nevertheless, the distribution of certain variables such as gender, age, and level of educational attainment is similar to the general sociodemographic profile of the Mexican elderly population [53]. In our follow-up, we experienced a loss of almost 25% of the sample, similar to that reported in other studies [42], [54]. To a great extent, this loss in follow-up came as a consequence of natural losses due to high mortality in this population group. Another limitation of our study is that our approach was limited to dementia syndrome diagnosis, without considering particular subtypes of dementia, which could show differential relationships with the NPSs [55]. Another limitation is that given the complexity of establishing the precise moment that dementia occurs, we have used an alternative method based on cumulative incidence ratios (over a 3-year follow-up). This method, although shown to be very robust, could overestimate the association, in comparison to the Cox proportional hazard ratio model, which may be more appropriate if the exact time of onset of dementia was assessed [29], [31].

Some strengths of this study are the following: (1) it was done with noninstitutionalized community sample of elderly people, contributing to an issue scarcely studied in low- and middle-income countries; (2) our study was able to identify clinical conditions such as dementia, mild cognitive impairment, diabetes, and cognitive function, all of which are considered risk or protective factors for developing dementia [3], [5], [17], [56], [57], [58], [59]; (3) we explored the associations of NPSs and incident dementia using multivariate techniques to limit confounders; and (4) during the construction of the proposed index, we included NPSs, cognitive, sociodemographic, and health variables, which are also considered risk factors, in one multivariable approach according to the classifications described by Tang et al. [52]. Another positive feature of this study is that our protocol allowed us to rule out primary psychiatric conditions as a cause of cognitive impairment and dementia. This is an important point when considering neuropsychiatric manifestations before dementia [23].

In conclusion, this report documents the association between the presence of delusions, hallucinations, depression, anxiety, and aberrant motor behavior, with the risk of developing incident dementia, independently of other known risk factors, including mild cognitive impairment. The study and development of new clinical indexes like those explored here, could be in the future a simple, low-cost strategy for screening population groups at dementia risk, particularly in environments with limited access to specialized services and sophisticated resources.

**Research in Context**

1. Systematic review: We searched PubMed and Scopus for articles in English or Spanish with the terms (“behavioral symptoms” OR “behavioural symptoms” OR “neuropsychiatric symptoms”) and (“predict*” OR “risk factor”) in title and/or abstract and “dementia” as MeSH term. Most reports that studied the relationship between neuropsychiatric symptoms and dementia (1) describe symptoms as sequelae of dementia (2) and those who treat them as predictors have been limited to analyzed isolated symptoms (such as depression or anxiety).
2. Interpretation: We show that old people with neuropsychiatric symptoms have more than twice the risk of developing dementia in the period of 3 years of follow-up, even after adjusting for cognitive and other variables of interest.
3. Future directions: Given the need to improve the ability to identify subjects at risk of developing dementia in the early stages and the complexity of integrating simple and inexpensive indicators, it is necessary to continue testing clinic markers that are easy to evaluate by general practitioners and trained health workers.
